# A dataset of forest biomass structure for Eurasia

**DOI:** 10.1038/sdata.2017.70

**Published:** 2017-05-16

**Authors:** Dmitry Schepaschenko, Anatoly Shvidenko, Vladimir Usoltsev, Petro Lakyda, Yunjian Luo, Roman Vasylyshyn, Ivan Lakyda, Yuriy Myklush, Linda See, Ian McCallum, Steffen Fritz, Florian Kraxner, Michael Obersteiner

**Affiliations:** 1 Ecosystems Services and Management Program, International Institute for Applied Systems Analysis, Laxenburg A-2361, Austria; 2 Forestry Faculty, Bauman Moscow State Technical University, Mytischi 141005, Russia; 3 Institute of Forest Siberian Branch Russian Academy of Sciences, Akademgorodok, Krasnoyarsk 66036, Russia; 4 Botanical Garden, Ural Division, Russian Academy of Sciences, Yekaterinburg 620144, Russia; 5 National University of Life and Environmental Sciences of Ukraine, Kyiv 03041, Ukraine; 6 Department of Ecology, School of Horticulture and Plant Protection, Yangzhou University, Yangzhou 225009, China; 7 Ukrainian National Forestry University, Gen. Chuprynka str. 103, Lviv 79057, Ukraine

**Keywords:** Forest ecology, Forestry

## Abstract

The most comprehensive dataset of *in situ* destructive sampling measurements of forest biomass in Eurasia have been compiled from a combination of experiments undertaken by the authors and from scientific publications. Biomass is reported as four components: live trees (stem, bark, branches, foliage, roots); understory (above- and below ground); green forest floor (above- and below ground); and coarse woody debris (snags, logs, dead branches of living trees and dead roots), consisting of 10,351 unique records of sample plots and 9,613 sample trees from ca 1,200 experiments for the period 1930–2014 where there is overlap between these two datasets. The dataset also contains other forest stand parameters such as tree species composition, average age, tree height, growing stock volume, etc., when available. Such a dataset can be used for the development of models of biomass structure, biomass extension factors, change detection in biomass structure, investigations into biodiversity and species distribution and the biodiversity-productivity relationship, as well as the assessment of the carbon pool and its dynamics, among many others.

## Background & Summary

Biomass is an important indicator of terrestrial vegetation and as such, is recognised as an Essential Climate Variable^1^ and an Essential Biodiversity Variable^2^. The link between biodiversity, tree species distribution and biomass^3^ as well as the biodiversity-productivity relationship^4^ are well recognised. Moreover, biomass is mentioned in six out of the seventeen UN Sustainable Development Goals^5^. Remote sensing is one of the most common approaches to estimate forest biomass and its dynamics over large areas. This includes measurements of canopy cover, vegetation status from different indexes, canopy height and forest structure^6,7^. However, there are no remote methods that can measure biomass density and the biomass structure by component, which can only be obtained from ground measurements. This is why field measurements are so crucial, i.e., they are the most accurate ways to learn about biomass structure, and they are needed to calibrate remote sensing instruments, model the carbon cycle, and assess forest productivity, among other uses.

Yet the sharing of biomass measurements has traditionally been highly problematic. Most researchers prefer to keep the raw data confidential and publish only the aggregated results or a limited number of the measured parameters. There are some reasons that can explain this situation. First of all, the destructive sampling method (DSM) for making biomass measurements on sample plots is a very labour-intensive process so the considerable investment needed over time does not incentivise researchers to share the data. Secondly, in some cases, agreements are made between researchers and the owners of the plots, which have tended towards closed use of the data by individual research projects. Finally, many experiments and measurements were undertaken in a pre-internet era and may not have not been published in English. Therefore, preserved in paper format in different countries around the world, these measurements have not been readily accessible to the scientific community.

**Figure 1:**
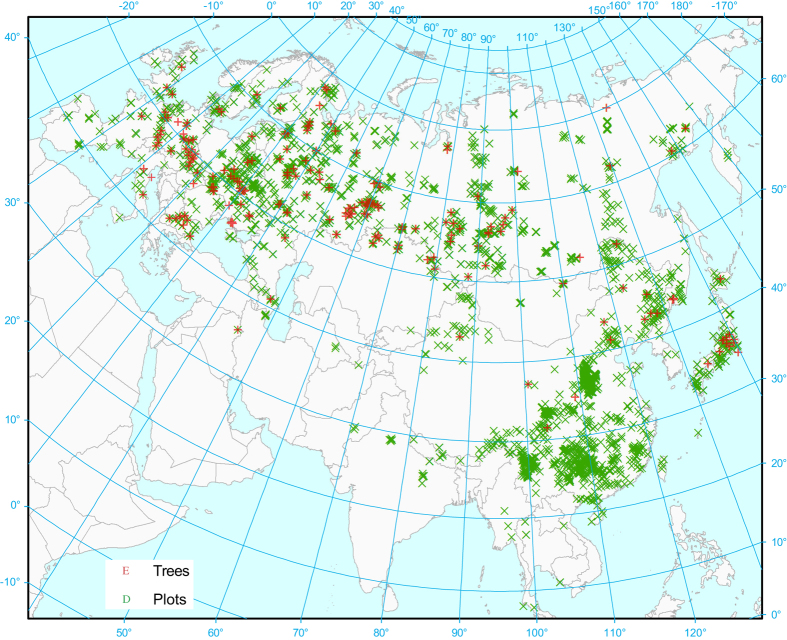
Location of sample trees and plots (Coordinate System: Asia north Albers equal area conic, central meridian 95° E).

To help remedy this situation, we have collected the most comprehensive dataset of *in situ* forest biomass measurements in Eurasia estimated by the DSM. The dataset has been compiled from a combination of experiments undertaken by the authors and from scientific publications. Every record contains an accompanying reference. The dataset consists of 10,351 sample plots and 9,613 sample trees (Fig. 1) from ca 1,200 experiments undertaken over the period 1930–2014. Note that these two tables are not completely linked but there is some overlap, i.e., 6,280 trees are associated with 791 plots. All other plots have no trees associated with them or vice versa.

The plot level dataset contains forest biomass structure per hectare, including live trees (stem, bark, branches, foliage, roots), understory (above- and below ground), green forest floor (above- and below ground) and coarse woody debris (snags, logs, dead branches of living trees and dead roots). Due to the compilation of quantities from diverse studies, some fractions (e.g., stem wood, foliage) are better represented than others (e.g., roots, green forest floor), which means that we reported only fractions where actual measurements were performed. In addition to biomass, we have recorded a number of other forest stand parameters where available, including tree species composition, average age, tree height, growing stock volume, etc. The tree level dataset consists of a description of the sample trees, their size and their biomass fractions (see Method section for more details).

The data presented here have been partly published before^8–14^, but never in a comprehensive, open access, electronic format that includes the full set of parameters. We have combined existing forest biomass datasets, removing duplicated records and merging complementary parameters to create a single fused product.

The dataset is complementary to existing datasets (e.g., refs 4,15) with almost no or little overlap observed. The dataset can be used for the development of models of biomass structure, allometric equations, biomass expansion factors (BEF), change detection of biomass structure, investigations into biodiversity and species distribution and the biodiversity-productivity relationship, and the assessment of the carbon pool and its dynamics, among others.

## Methods

All the data presented here were collected by the DSM. The background prerequisite of the method is to follow the major requirements of a statistically sound sampling procedure. Sample plots should be representative of the selected forest unit and include 200–300 trees. Within sample plots, the diameter at breast height (DBH, which is usually at 1.3 m or 4.5 feet) is measured for each tree. The measurements of tree height are provided for 12–15 trees by species, selected proportionally to the number of trees by diameter class in order to develop height-diameter regression relationships. These and other reported results of the measurements allow for the estimation of basic biometric (mensuration) characteristics of stands such as tree species composition, age, average diameter and height, growing stock volume, etc. For the assessment of live biomass, a number of trees are selected, cut and measured, which is outlined in more detail as follows:

- The sample trees selected for destructive measurements (typically 5–15 per sample plot) should represent all tree species and the full variety of tree diameters at the sample plot.
- Trees are cut and measured for as accurate an estimation as possible of taper, age, volume, increment and other biometric characteristics.
- The wood and bark are sampled 5–10 times at different heights for every sample tree (usually as cross-sections of 2 to 3 cm in width).
- The crown of the sample trees is sampled to represent all the parts from the bottom to the top including the full range of branch sizes for further analysis (separating foliage and drying) as well as weighing, in both the fresh and oven dry states.
- Leaf area index was calculated through the size-to-mass ratio of the sample of foliage, upscaled to 1 ha with foliage biomass.
- In order to measure root biomass, soil sampling is employed to represent different distances from the stem and different depths^10^. The samples are washed with water in order to extract the roots, which are separated by whether they are dead/alive, tree/grass and by size. However, most of the field studies omit below ground investigations due to the very high labour-consuming nature of this work.
- The understory is accounted for in sub-plots usually of 2×2 m, regularly distributed over the plot. In the case of an unequal distribution of understory, mapping of the canopy windows is then recommended with a separate understory accounting in these windows and under the canopy. The numbers by plant species and height are recorded. The average representatives of each species and height class are harvested for further separation by biomass fraction, drying and weighing.
- The green forest floor is described and sampled for subsequent analysis at 1×1 m sub-plots.
- Coarse woody debris is accounted for by type (logs and snags), size (length and diameter), and the stage of decomposition, and is sampled accordingly.

The sampled patterns are delivered to laboratories, oven dried and weighed. The results are recorded in units of mass of dry matter. The methods mentioned above are described in detail in a number of publications, e.g., refs 10,16,17.

The data collected through the DSM can be found in Biomass_plot_DB.xlsx (plot data, Data Citation 1) and Biomass_tree_DB.xlsx (tree data, Data Citation 2).

## Data Records

A list containing the fields and summary statistics is presented for the sample plot (Table 1) and tree (Table 2) datasets.

Sample plot data can be found in Biomass_plot_DB.xls (Data Citation 1).

Sample tree data can be found in Biomass_tree_DB.xls (Data Citation 2).

**Figure 2:**
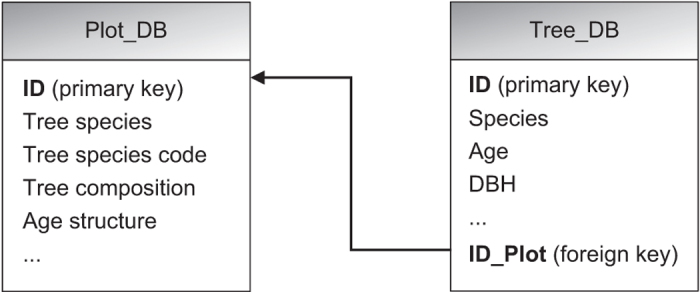
Linkage between the plot and tree datasets.

The linkage between the two tables is shown in Fig. 2.

## Technical Validation

The dataset represents a range of countries (Table 3), biomes (Table 4) and tree species (Table 5). The most representative countries are Russia, Ukraine, China, and Kazakhstan (Table 3).

Most of the observations fall in the Boreal and Temperate biomes (Table 4).

Pine forests have been sampled the most with 44% of the records (Table 5) followed by spruce (12%), birch (10%), larch (6%), poplar (5%) and oak (5%).

The DSM remains the most labour-costly and precise method of assessing forest biomass. The accuracy of the method and, consequently, the reliability of the presented biomass data depend on the number of sample trees. The error of the method has been estimated and documented in several studies^10,18–20^, in which sub-samples of the data were made from a comprehensive dataset, e.g., the entire harvest of all trees at the sample plot to investigate how the accuracy changed with sample size. The results show that the accuracy varies depending on the type of biomass parameter considered, i.e., the most reliable variable is the estimation of stem biomass (92–94%) while the least reliable are the crown (80–90%) and belowground (70–80%) biomass estimates.

**Figure 3:**
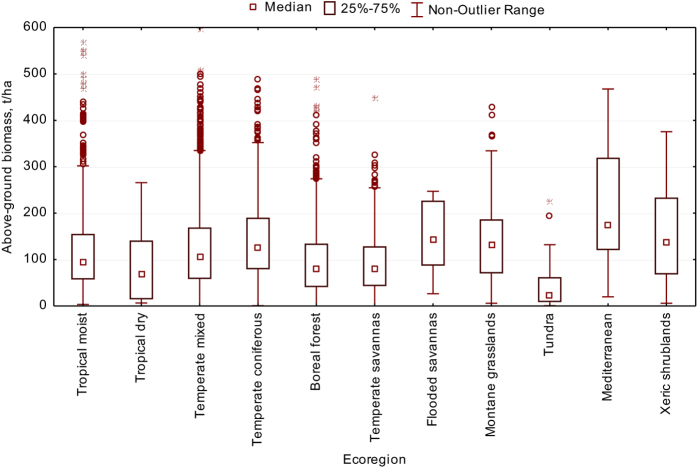
Above ground biomass by biome.

We have provided a validation of the data by checking their consistency with expected ranges for these parameters. The distribution of forest biomass by major biomes is provided in Fig. 3, which shows reasonable variation with climatic condition.

**Figure 4:**
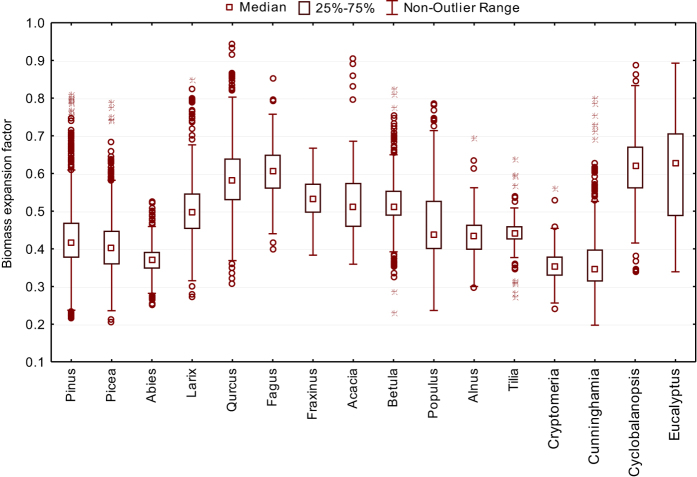
Ratio of stem biomass to its volume for different tree genera.

**Figure 5:**
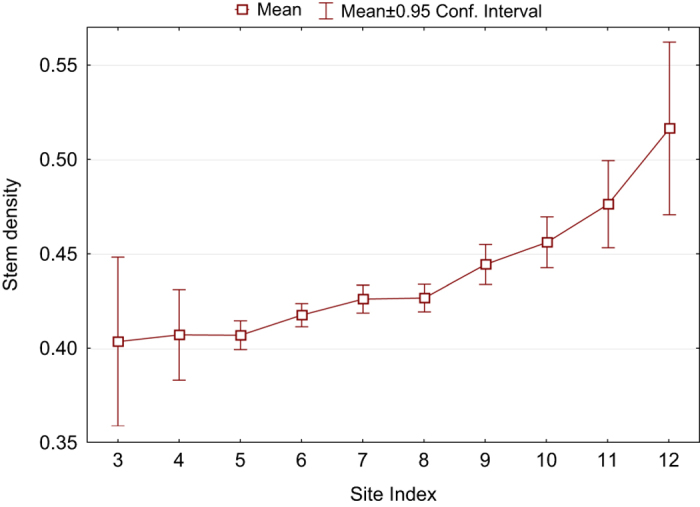
Ratio of stem biomass to its volume for different site indexes of pine stands. Site index 3 refers to an average stand height of 38.2–41.8 m at the age of 100 years old, while a site index of 12 refers to 4.8–8.4 m at the same age.

Relative indicators (especially BEF) are usually the most useful for validation. For example, wood density varies substantially with tree species (Fig. 4) and site index (Fig. 5), but stays within the expected range reported in a number of ecological publications (i.e., ref. 15).

**Figure 6:**
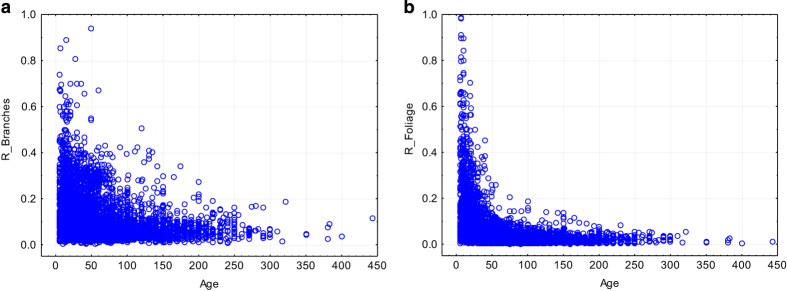


Figure 6 illustrates that the share of the crown biomass depends very much on the stand age, which is the expected relationship (e.g., refs 10,21).

**Figure 7:**
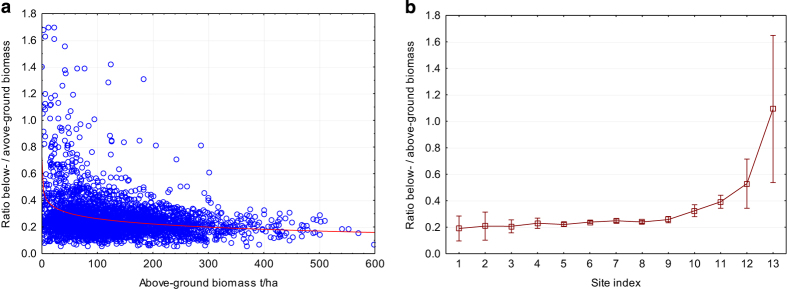
Ratio of belowground to aboveground biomass, which depends on the (a) aboveground biomass value or (b) the site index.

The distribution of the belowground live biomass is shown in Fig. 7. A larger below ground biomass share is typically observed in low biomass forests and/or tough site conditions.

**Figure 8:**
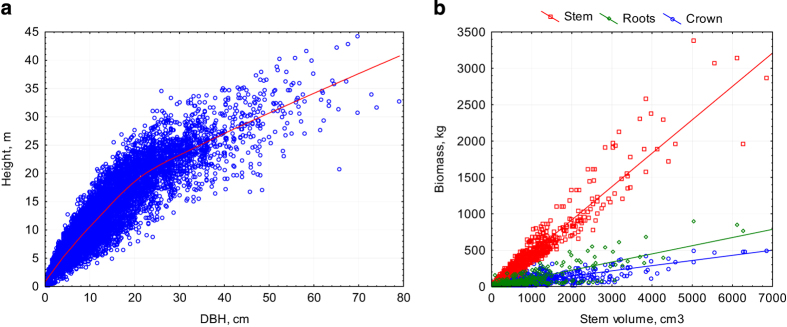


Some common relationships in the sample tree parameters are presented in Fig. 8. The outliers can be explained by the individual characteristics of the tree species and the climate gradient.

With respect to geographic and parametric representations, the data cover the forests of the major forest-forming species of Eurasia in a satisfactory way. The outliers (i.e., values outside the limits of an average of ±3 s.d.’s) are negligible but where present, they can usually be explained by age, site or climatic conditions, as well as by tree species. Overall the data demonstrate satisfactory consistency with reported ranges of national and zonal aggregations and regulations (e.g., yield tables^22^).

## Usage Notes

The data are stored in Excel xlsx format. Sheets ‘Plot_db’ and ‘Tree_db’ contain the data records. The sheet ‘Species’ lists the tree species code, and the English and Latin names. The sheet ‘References’ contains a reference for every individual data record. The sheet ‘Field description’ describes the dataset fields and the data units.

The dataset can be used for a number of applications, but are not limited to the following examples. An early version of the dataset was used to develop models of biomass structure^22,23^, where the components of biomass (stem, branches, foliage, roots) were shown to be a function of age, site index, relative stocking and growing stock volume. Similar data collections for different regions have been utilised to derive BEF (e.g., ref. 24) and allometric equations (e.g., ref. 25). When the dataset contains long term measurements, there is then the possibility to track structural changes in the biomass (e.g., ref. 26).These types of data are also crucial for the assessment of the carbon pool and its dynamics (e.g., ref. 27). These data are also useful in biodiversity studies, e.g., to investigate relationships between biodiversity and species distribution (e.g., ref. 3), and the biodiversity-productivity relationship (e.g., ref. 4). Note that the data presented here are not suitable for the direct calibration/validation of products derived from remote sensing, because sample trees may have been cut down and the precision of the geographic coordinates, in many cases, does not allow for precise enough geolocation.

## Additional Information

**How to cite this article:** Schepaschenko, D. *et al.* A dataset of forest biomass structure for Eurasia. *Sci. Data*. 4:170070 doi: 10.1038/sdata.2017.70 (2017).

**Publisher’s note:** Springer Nature remains neutral with regard to jurisdictional claims in published maps and institutional affiliations.

## Supplementary Material



## Figures and Tables

**Table 1:** Sample plot dataset: a list of the fields and descriptive statistics.

**Field**	**Number**	**Unique**	**Median**	**Min**	**Max**
*Forest mensuration parameters:*					
Dominant tree species (Latin name)	10,351	296			
Dominant tree species code (see list of the codes in ‘Species’ sheet of the excel file)	10,351	28			
Tree species composition (percentage of tree species volume according to its contribution to the total stand growing stock volume)	9,819				
Age structure of stands (even- or uneven-aged)	700	2			
Origin (natural or planted)	9,860	2			
Site index^22^ (0 means that the forest can reach 49–53 m height at 100 years old while a value of 12 means that the stand will only reach a height of 5–8 m)	7,323		7	0	12
Mean stand age (year)	10,178		40	1	443
Average height of the stand—height of a tree with average DBH on the plot, obtained from the diameter-height curve (m)	8,143		13.8	0.1	65.1
Average DBH—diameter at breast height (1.3 m above ground) (cm) calculated as the quadratic mean of the DBH of individual trees	7,993		14.3	0.1	114
Number of trees per hectare	9,403		1,483	7	10,530,000
Relative stocking—ratio of basal area of a plot to basal area of the ‘normal stand’—ideal stands due to national standards (typically from 0 to 1)^22^	5,625		0.9	0.1	2.0
Basal area—total cross-sectional area of live trees at breast height in a plot (m^2^ ha^−1^)	1,075		24.2	0.1	57.7
Growing stock volume—volume of stems of all living trees (m^3^ ha^−1^)	9,590		170	0.0	3,831
*Live biomass (t oven dry matter ha*^*−1*^)					
Stem over bark	9,536		76.2	0.0	1,280.3
Bark of stem	4,252		9.0	0.0	74.7
Crown wood over bark	9,521		12.1	0.01	214.0
Leaves & needles	9,882		5.1	0.01	103.5
Stump & roots of trees	4,033		24.4	0.00	242.0
Fine roots with several thresholds: 1, 2, 5 and 7 mm by diameter	11		0.36	0.08	1.85
Undergrowth & shrubs above ground	675		1.1	0.0	73
Undergrowth & shrubs including roots	282		1.9	0.0	78.7
Green forest floor above ground	2,092		1.1	0.0	65.1
Green forest floor including roots	632		1.8	0.0	35.2
*Dead organic matter (t oven dry matter ha*^*−1*^):					
Snags	114		2.9	0.04	74.6
Logs	37		4.3	0.5	74.4
Dead branches of living trees	512		2.0	0.02	79.0
Dead roots	65		1.6	0.01	35.3
Litter	1,039		7.5	0	193.1
*General info:*					
Country code (ISO ALPHA-3)	10,351	43			
Latitude (8 N to 72 N)	10,351		51.3	7.6	72.5
Longitude (8 W to 160 E)	10,351		70.2	−45.9	160.7
Altitude (m a.s.l.)	3,450		600	0	4,240
Year of measurement	9,092		1,986	1,875	2,014
Number of trees selected for destructive sampling	1,709		5	1	102
Leaf area index of trees	1,303		7.3	0.2	41.8
Ecoregion^28^	10,351	123			
Reference	10,351	1,482			

**Table 2:** Sample tree dataset: a list of the fields and descriptive statistics.

***Field***	**Number**	**Unique**	***Median***	**Min**	**Max**
*Sample tree description and size:*					
Tree species (Latin name)	9,613	90			
Age (years)	9,575		36	3	430
Diameter at breast height—DBH (cm)	9,518		12	0	98.0
Height of the tree (m)	8,625		11.6	0.1	44.2
Height to crown base (m)	5,774		5.3	0	25.8
Diameter (maximal) of the crown (m)	4,091		2.2	0	14.3
Stem over bark volume (dm^3^)	7,169		68.4	0	6,984
Stem bark volume (dm^3^)	5,404		11.0	0	678.0
Origin (natural or planted)	9,530	2			
*Live biomass (kg oven dry matter*^*1*^)					
Stem over bark	7,466		29.4	0	4,122.0
Bark of stem	4,799		4.9	0	280.0
Crown wood over bark	8,862		3.9	0	1,091.8
Leaves & needles	8,896		2.0	0	305.0
Above ground	7,474		36.6	0	5,089.0
Stump & roots of trees	1,746		5.0	0	901.0
Total tree	1,712		28.8	0	5,134.8
*Location and reference*					
Country code (ISO ALPHA-3)	9,613	21			
Latitude	9,613		54.7	31.5	69.9
Longitude	9,613		55.8	−2.5	155.0
Altitude (m a.s.l.)	226		162	45	3,620
Number of trees per hectare	8,592		2,003	69	900,000
Reference	9,613	159			
Notes	430				
Ecoregion^28^	9,613	48			
Link to the sample plot (ID of the sample plot dataset)	6,280	791			

**Table 3:** Distribution of records by countries.

**Country name**	**Plots**	**Trees**
Belgium	34	14
Belarus	439	8
Bulgaria	75	137
China	2,933	65
Czech Republic	20	153
Denmark	85	1
Finland	38	—
France	63	24
United Kingdom	97	42
Germany	184	167
Hungary	28	9
India	37	—
Italy	29	9
Japan	412	186
Kazakhstan	393	1,564
Lithuania	33	—
Mongolia	10	57
Russian Federation	4,228	4,771
Slovakia	22	22
Sweden	56	—
Switzerland	6	136
Ukraine	897	2,238
Other countries	232	10
Total	10,351	9,613

**Table 4:** Distribution of dataset records by biomes.

**Biome**^28^	**Plots**	**Trees**
Tropical and Subtropical Moist Broadleaf Forests	1,197	—
Tropical and Subtropical Dry Broadleaf Forests	6	—
Temperate Broadleaf and Mixed Forests	4,673	2,788
Temperate Coniferous Forests	655	606
Boreal Forests/Taiga	2,072	2,805
Temperate Grasslands, Savannas, and Shrublands	1,385	3,264
Flooded Grasslands and Savannas	39	1
Montane Grasslands and Shrublands	171	18
Tundra	67	123
Mediterranean Forests, Woodlands, and Scrub	55	8
Deserts and Xeric Shrublands	31	—

**Table 5:** Distribution of the number of records by tree genus.

**Tree genus**	**Sample plots**	**Sample trees**
Abies	322	386
Acacia	41	—
Acer	7	27
Alnus	142	31
Betula	653	1,291
Carpinus	16	22
Castanopsis	52	—
Chamaecyparis	38	10
Chosenia	6	17
Cryptomeria 1	97	29
Cunninghamia 2	506	—
Cupressus	31	—
Cyclobalanopsis 3	249	—
Eucalyptus 4	94	—
Fagus	220	56
Fokienia	33	—
Fraxinus	55	31
Larix	712	566
Picea	1,067	1,298
Pinus	4,043	4,668
Populus	530	513
Pseudotsuga	27	13
Quercus	805	130
Robinia	40	24
Salix	22	23
Sorbus	—	20
Tilia	267	402
Other species	276	56
Total	10,351	9,613

## References

[d1] PANGAEA Schepaschenko D. 2017 https://doi.pangaea.de/10.1594/PANGAEA.871465

[d2] PANGAEA Schepaschenko D. 2017 https://doi.pangaea.de/10.1594/PANGAEA.871491

